# Eat More Healthily: Nutrition Quality and Nutrition Information of Foods

**DOI:** 10.3390/nu17030590

**Published:** 2025-02-06

**Authors:** Ana B. Ropero

**Affiliations:** Institute of Bioengineering, Miguel Hernández University, 03202 Elche, Spain; ropero@umh.es

Healthy eating is one of the main factors that contribute to reducing the risk of noncommunicable diseases [1]. Governments are partly responsible for protecting citizens against the risk posed by unhealthy diets. One of the main initiatives is to provide nutritional information about food to consumers. It is mandatory to display the nutrition declaration on foods in more than 90 countries around the world [2,3]. Evidence from randomized controlled trials (RCTs) suggest that nutrition declaration likely improves consumer understanding of the nutritional quality/content of foods [4]. However, previous reviews had reported that consumer understanding of the quantitative information present in labels is limited [5]. To overcome these difficulties, simpler information may be required, such as front-of-pack labelling (FOPL), which is already used in some countries [3,6,7].

According to the World Health Organization, “the principal aim of FOPL is to provide convenient, relevant and readily understood nutrition information or guidance on food packs, to assist all consumers to make informed food purchases and healthier eating choices” [6]. A recent review of 221 articles concluded that “FOPL likely improved consumer understanding of the nutritional quality/content of foods (moderate certainty of evidence), and the healthfulness of food choices (moderate certainty) and purchases (moderate certainty). Interpretive FOPL had a greater effect on these outcomes compared with noninterpretive systems (moderate certainty). There was inconsistency in the best-performing interpretive FOPL system” [4]. The effects observed were small [4].

Nutrition and health claims also provide useful food information to consumers. They are regulated in more than 70 countries, mainly in Europe and America [3]. Manufacturers often use them to highlight one or more nutritional characteristics of interest. According to the Codex Alimentarius, “nutrition claim means any representation which states, suggests or implies that a food has particular nutritional properties including but not limited to the energy value and to the content of protein, fat and carbohydrates, as well as the content of vitamins and minerals” [8]. Regarding health claims, they mean “any representation that states, suggests, or implies that a relationship exists between a food or a constituent of that food and health” [8]. The effect of nutrition and health claims on the perceived healthiness of foods has been extensively studied. A recent systematic review concluded that RCT evidence “suggests that claims likely increased consumer perceptions of food healthfulness and increased choice and purchases of labeled foods (both moderate certainty), irrespective of nutritional quality” [9]. However, the evidence on the effect on diet is of very low certainty [9]. Some studies have reported that foods with nutritional and/or health claims tend to have better nutritional quality than those without these claims [10,11,12,13]. However, this is not always the case [14]. In addition, the results largely depend on the criteria used to assess the nutritional quality [12]. Despite potential improvements, foods with NC/HC are not necessarily healthy.

This Special Issue has important contributions to these and related subjects, which are summarized below.

## 1. Parents’ Opinions on Sugary Drink Warning Labels

Consumers’ perception and interpretation of FOPL determine their effectiveness. Negative reactions to FOPL may be caused by the disassociation between the intended use and the way consumers interpret and use them. For this reason, the paper by Miller et al. is of importance (contribution 1). The authors present a preliminary study of parents’ opinions on several options for sugary drink warning labels. Parents highlighted some characteristics of the labels, such as credibility, being easy to understand, relevant and informative. The authors concluded the following: “the label that was perceived the most favourable was the text label depicting the number of teaspoons of sugar in beverages”. Interestingly, the authors tested for possible negative reactions, which is unusual in this type of studies. They observed that some of the labels were perceived as having negative implications. Participants raised some issues, which would be interesting to evaluate in the full-scale study. The authors may be able to determine whether these issues are related to the educational level of the participant parents.

## 2. Consumer Awareness of Food Processing and the Association with Healthiness

Ultra-processed (UPF) foods have been in the spotlight in recent years because of their association with health, such as an increased risk of chronic diseases, cancer and mortality [15,16]. The NOVA classification adopted by FAO has been the focus of recent controversy [17,18]. The bottom line is that UPFs include food groups with different associations with health [17]. This is a major drawback to providing easy and straightforward recommendations to the general population. The consequence is that the average consumer would find it difficult to choose “good” UPFs over the “bad” UPFs. Somehow, Bolhuis et al. anticipated this issue and conducted a pilot study with consumers in three countries (Netherlands, Italy and Brazil) (contribution 2). The authors used Nutri-Score to classify UPF foods as “not healthy” or “healthy” (scores D or E and A or B, respectively). Consumers rated “not healthy” UPF correctly. However, they had difficulties in rating the “healthy” UPFs because many of them were classified as “not healthy”. Yet, the authors were positive and concluded the following: “these preliminary findings suggest that consumers incorporate, to some extent, the degree of industrial processing while assessing the healthiness of food products”.

## 3. Nutritional Description of Organic and Conventional Food Products

Health is one of the main reasons why consumers choose organic food over conventional food [19]. They think that organic foods have less exposure to contaminants and higher nutritional value [19]. Some studies have shown a higher micronutrient and antioxidant content in natural organic foods compared to conventional foods [19,20,21]. However, some studies failed to show nutritional differences [22]. As for processed foods, only a few studies have been published, either with small samples or with very heterogeneous food groups. Ropero et al. conducted a comprehensive analysis of large samples of six specific food types (contribution 3). The study includes nutrient composition and assessment of nutritional quality, as well as the use of nutrition claims and fortification (contribution 3). The main conclusion of the analysis is that “consumers’ perception that organic food products are healthy is unfounded from a nutritional point of view”. In fact, only one in five organic foods is classified as ’healthy’. Organic foods use nutrition claims more often than the conventional version, while only a few of them were fortified with vitamins or minerals.

## 4. Nutritional Description of Processed Foods with Fibre-Related Nutrition Claims

Studies have shown that consumers have positive attitudes towards fibre-related nutrition and health claims [23,24,25,26,27]. However, only a few studies have analyzed the nutritional quality of foods with fibre-related nutrition claims [10,28,29]. These are limited in terms of the scope of the analysis or the type of foods included. In this Special Issue, Ropero et al. published a comprehensive nutritional analysis of foods with fibre-related nutrition claims available on the Spanish market (contribution 4). The authors also studied the rate of micronutrient fortification, as well as the proportion of organic foods. The results show that most foods with fibre-related claims were classified as “less healthy”. Unsurprisingly, these foods had more fibre, but they were not nutritionally better than those without these claims. The prevalence of organic versions was lower, while similar rates of micronutrient fortification were obtained compared to foods without fibre-related claims.

## 5. Nutritional Quality of Chain Restaurant Menu Items in Ontario

While the use of nutrition labelling of foods available in supermarkets is common, its use in restaurants is rare. Restaurant foods are associated with excessive energy intake and poor nutritional quality [30,31]. Therefore, consumers would benefit greatly from the use of nutrition labelling in restaurants. To this end, the healthy Menu Choices Act 2015 was enacted in 2017 in Ontario [32]. Since then, the display of the energy content of food in menus is mandatory in food service establishments with 20 or more outlets [32].

In this Special Issue, Yang et al. compared the nutritional quality of food from regulated and unregulated restaurants (contribution 5). The main conclusions of the study were that “menu items from regulated restaurants had smaller serving size, lower levels of energy and nutrients of public health concern compared to those from the unregulated restaurants, suggesting potential downstream beneficial effects of menu labelling in lowering caloric content and nutrients of public health concern in foods”.

## 6. Consumers and the Use of Food Labels in Jordan

Consumers’ understanding of food labels is crucial to developing the most effective in improving the diet of the general population. To this end, it is recommended to conduct studies with consumers before establishing regulations on the food information to be provided. In addition, once the regulation is in force, it is of utmost importance to analyze its efficacy as well as consumers’ understanding. Comprehension of food labels depends on many factors, including sociodemographic factors and lifestyle choices [33,34]. The culture and tradition of the country may also be contributing factors and fully justify conducting consumers studies in different countries.

Rashaideh et al. analyzed Jordanian consumers’ comprehension of food labels, their attitudes and their use (contribution 6). Their main conclusion is that “comprehensive users of food labels (26.4%) were more likely female, responsible for grocery shopping, and had higher mean knowledge and attitude scores. Jordanian consumers seem to have good practices and attitudes related to food label use but suboptimal knowledge regarding content”.

## 7. Dietary Food Supplements in European National Nutrition Surveys

The National Nutrition Surveys are of great importance to determine the possible nutritional deficiencies of the population and to establish regulations to minimize them. The use of dietary supplements (DSs) has been on the rise in recent years [35]. DSs might be recommended to certain high-risk subpopulations, such as women before conception and in the first trimester of pregnancy [36]. However, the use of DSs extends beyond that, as many consumers rely on them to meet recommended dietary allowances. Therefore, National Nutrition Surveys should record the use of DSs. Papatesta et al. analyzed this in 53 European participants and only 21 of them had data available at that time (contribution 7). The authors described DS consumers in detail by country, gender, age, education, BMI, physical activity, body mass index, health status, types and number of DSs. They also analyzed the contributions of DSs to diet.

## 8. Compliance with Brazilian Front-of-Pack Labeling

Brazil enacted a regulation regarding the use of FOPL in 2020 and it was fully implemented in 2022 [37]. This regulation establishes that warning labels are mandatory on foods with a high content of sugar, saturated fat or sodium content according to the criteria defined in Normative Instruction No. 75/2020 [37]. Senda et al. conducted an analysis on the implementation of this regulation in the Brazilian market in 2023 (contribution 8). They evaluated 2145 foods and only 541 of them had warning labels. Nearly one in four foods were high in added sugar, while almost one in three were also high in saturated fat. The authors argued that the infrequent use of warning labels may have been due to reformulation by the food industry. They also suggested that they may not have had time to adapt to this regulation because most of the foods have a very long shelf life.

## 9. Nutritional Quality of Chinese Processed Meat Products

According to the World Health Organization, “Nutrient profiling is the science of classifying or ranking foods according to their nutritional composition for reasons related to preventing disease and promoting health” [38]. Nutrient profile models (NPMs) may be used for a variety of purposes, such as restricting marketing of unhealthy foods, applying food taxes, or FOPL [38]. Harmonized NPMs do not exist and many have been developed over the last decade [39]. They may vary greatly and thus result in important divergences when applied to foods.

Ding et al.’s contribution focused on processed meat in China (contribution 9). The authors used the Chilean FOPL and the Chinese Healthier Choice Logo [40,41]. The Chilean criteria are universal for all solid foods, regardless of food categories. However, the Chinese criteria are food-type specific. When both were applied to processed meat products available in China, more foods met the Chinese criteria than the Chilean FOPL. The main reason may have been that the Chinese threshold for sodium is higher than in the Chilean FOPL. The difference in the threshold for total sugar is of little importance for this type of food. Another difference between the two NPMs is that energy is not taken into account in the Chinese NPM, but instead a maximum is set for total fat [40,41].

## 10. Nutri-Score Applied to Meat, Fish, and Dairy Alternatives

Nutri-Score is the FOPL used in several countries in Europe [7]. The purpose of Nutri-Score is to compare foods of the same type and it results in a classification according to five colours [42]. Nutri-Score was first implemented in France in 2017 as a modification of the NPM developed by the Food Standards Agency in 2004–2005 [42]. It has been adopted by Portugal, Spain, Austria, Belgium, Germany, Luxembourg and Switzerland over the years. The Scientific Committee proposed an update of the algorithm for solid foods in 2022 and for beverages in 2023 [43,44]. These updates were adopted and applied in 2024 [42]. Several important changes were made, such as evaluating the presence of sweeteners in beverages or reducing tolerance to sugar and sodium/salt [43,44].

In this Special Issue, Huybers and Roodenburg applied both algorithms to meat, fish and dairy alternatives (contribution 10). They reported that the proportions of foods classified as A and B were lower with the new algorithm. In addition, the nutritional composition of meat and milk alternatives with labels A and B improved. The authors concluded that “overall, the new Nutri-Score algorithm is more in line with the Dutch dietary guidelines for plant-based meat and dairy alternatives, though challenges remain with respect to micronutrient (iron, calcium, vitamin B12), salt, and protein content”.
